# Identification of women with early breast cancer by analysis of p43-positive lymphocytes

**DOI:** 10.1038/sj.bjc.6690434

**Published:** 1999-05

**Authors:** L Auerbach, M Hellan, M Stierer, A C Rosen, C Ausch, R Obwegeser, E Kubista, G Wolf, H R Rosen

**Affiliations:** Departments of Gynaecology and Obstetrics and Diagnostic Radiology, and Clinic for Blood Group Serology, Division of Special Gynecology, Währinger Gürtel 18–20, Vienna, 1090, Austria; Department of Surgery, Hanusch Medical Center, Heinrich Collin Str. 30, Vienna, 1140, Austria; Departments of Gynaecology and Obstetrics and Department of Surgery, Ludwig Boltzmann Research Institute for Surgical Oncology, Langobardenstr. 122, Vienna, A-1220, Austria

**Keywords:** breast cancer, flow cytometry, lymphocytes, p43, T-cells

## Abstract

Regular screening mammographies and increasing knowledge of high-risk groups have resulted in an improvement in the rate of detection of smaller malignant lesions. However, uncertain minimal mammographic features frequently require further costly and often uncomfortable investigation, including repeat radiological controls or surgical procedures, before cancerous lesions can be identified. Placental isoferritin (p43), a protein with immunosuppressive effects, has been detected on the surface of lymphocytes taken from peripheral blood in patients with breast cancer. In this study we evaluated the sensitivity and specificity of the expression of p43-positive lymphocytes as a marker in early stage breast cancer and also investigated its expression on T-cell subpopulations. The presence of p43-positive lymphocytes was investigated using the monoclonal antibody CM-H-9 and flow cytometry in 76 women with controversial, non-palpable mammographic findings who were undergoing surgical biopsy. Patients with early breast cancer (*n* = 48) had significantly higher p43-positive cell values (median 3.83%, range 0.98–19.4) than patients with benign lumps (*n* = 28, median 1.43%, range 0.17–3.7) or controls (*n* = 22, median 1.3%, range 0.4–1.87) (*P* < 0.0001). At a cut-off level of 2% p43-positive cells a sensitivity of 91.7% and a specificity of 89.3% for detection of breast cancer could be reached. While the median ratio of total CD4+/CD8+ cells was 2.6, a ratio of 1.3 was found for the p43-positive subpopulation (*P* < 0.001), thus indicating a significant link between p43 and CD8+ cells. The determination of p43-positive lymphocytes in peripheral blood could serve as an additional diagnostic tool in patients with controversial mammographic findings and could also reduce the need for cost-intensive and often uncomfortable management of these patients. © 1999 Cancer Research Campaign


*British Journal of Cancer* (1999) **80**(5/6), 874–878
© 1999 Cancer Research Campaign
Article no. bjoc.1998.0434

### Identification of women with early breast cancer by analysis of p43-positive lymphocytes

**L Auerbach[1], M Hellan[1], M Stierer[4], AC Rosen[5], C Ausch[6], R Obwegeser[1], E Kubista[1], G Wolf[2], HR Rosen[6] and S Panzer[3]**

Departments of [1]Gynaecology and Obstetrics and [2]Diagnostic Radiology, and [3]Clinic for Blood Group Serology, Division of Special Gynecology, University of Vienna Medical School, Währinger Gürtel 18–20, 1090 Vienna, Austria, [4]Department of Surgery, Hanusch Medical Center, Heinrich Collin Str. 30, 1140 Vienna, Austria; Departments of [5]Gynaecology and Obstetrics and [6]Ludwig Boltzmann Research Institute for Surgical Oncology, Department of Surgery, Danube Hospital/SMZ-Ost, Langobardenstr. 122, A-1220 Vienna, Austria

**Summary** Regular screening mammographies and increasing knowledge of high-risk groups have resulted in an improvement in the rate of detection of smaller malignant lesions. However, uncertain minimal mammographic features frequently require further costly and often uncomfortable investigation, including repeat radiological controls or surgical procedures, before cancerous lesions can be identified. Placental isoferritin (p43), a protein with immunosuppressive effects, has been detected on the surface of lymphocytes taken from peripheral blood in patients with breast cancer. In this study we evaluated the sensitivity and specificity of the expression of p43-positive lymphocytes as a marker in early stage breast cancer and also investigated its expression on T-cell subpopulations. The presence of p43-positive lymphocytes was investigated using the monoclonal antibody CM-H-9 and flow cytometry in 76 women with controversial, non-palpable mammographic findings who were undergoing surgical biopsy. Patients with early breast cancer ($n$ = 48) had significantly higher p43-positive cell values (median 3.83%, range 0.98–19.4) than patients with benign lumps ($n$ = 28, median 1.43%, range 0.17–3.7) or controls ($n$ = 22, median 1.3%, range 0.4–1.87) ($P$ < 0.0001). At a cut-off level of 2% p43-positive cells a sensitivity of 91.7% and a specificity of 89.3% for detection of breast cancer could be reached. While the median ratio of total CD4+/CD8+ cells was 2.6, a ratio of 1.3 was found for the p43-positive subpopulation ($P$ < 0.001), thus indicating a significant link between p43 and CD8+ cells. The determination of p43-positive lymphocytes in peripheral blood could serve as an additional diagnostic tool in patients with controversial mammographic findings and could also reduce the need for cost-intensive and often uncomfortable management of these patients.

**Keywords:** breast cancer; flow cytometry; lymphocytes; p43; T-cells

Early detection of breast cancer significantly improves prognosis. Increasing knowledge of high-risk groups and regular screening mammography have resulted in an improvement in the rate of detection of smaller malignant lesions (Arnesson et al, 1995; Tabar et al, 1995). However, certain minimal mammographic features, such as small vague densities, indefinable microcalcification or subtle architectural distortions, alone or in combination, can also represent non-specific indications of cancerous lesions (Sickles, 1994; Ciatto et al, 1995; Hiatt and Pasick, 1996; Maes et al, 1997). In addition, the diagnostic accuracy of mammographic screening in women under 50 with dense breast parenchyma has been questioned by some authors (Black et al, 1995; Leitch, 1995). For this reason, uncertain mammographic findings sometimes require further costly and often uncomfortable investigations, such as including repeat radiological controls, magnetic resonance imaging (MRI), positron emission tomography (PET) or invasive surgical procedures (Ciatto et al, 1995; Harris and Leininger, 1995; Lidbrink et al, 1996; Flanagan et al, 1998; Friedrich, 1998). A reliable diagnostic marker would therefore be useful to differentiate between women with early-stage breast cancer and women with benign breast lesions.

*Received 22 July 1998*
*Revised 7 December 1998*
*Accepted 9 December 1998*

*Correspondence to:* HR Rosen

Moroz and co-workers identified an isoform of placenta-derived ferritin consisting of a specific 'super' heavy (43-kDa) chain (p43) (Moroz et al, 1985). This isoferritin has been shown to mediate immunosuppression during pregnancy (Sirota et al, 1989; Maymon and Moroz, 1996). Moreover, an elevated p43 level has been observed in the peripheral blood of patients with Hodgkin's disease, AIDS and breast cancer (Moroz et al, 1977, 1989; Rosen et al, 1992). In an investigation by Moroz involving more than 3000 women, 80% of stage I and II breast cancer patients, but less than 10% of women with benign breast disease or healthy controls, had increased p43 expression on lymphocytes, as determined by radioimmunoassay (Moroz et al, 1989).

So far, only a few studies have examined a possible effect of p43 expression on the immune system of breast cancer patients, and thus on its role in the pathomechanism of cancer development (Bilik et al, 1989; Fargion et al, 1991; Rosen et al, 1996). In-vitro studies demonstrated the immunosuppressive action of p43 on lymphocytes (Reinerova et al, 1993; Sedlak et al, 1995).

Our study focused initially on determining the expression of p43 in a highly selected patient population with uncertain mammographic findings and non-palpable breast lesions, who were recruited from a breast cancer screening programme. By distinguishing between the expression of p43 on CD4+ and CD8+ cells, respectively, in benign and malignant breast lesions, we sought to identify the lymphocyte subpopulation predominantly expressing p43.

### MATERIALS AND METHODS

A total of 76 consecutive female patients were scheduled for surgical biopsy because of uncertain mammographic findings. All patients were seen for the first time at a breast cancer clinic at one of the three participating centres. Inclusion criteria were non-palpable mammographic lesions in accordance with the characteristics listed in Table 1. All of these radiological criteria for potential malignancy had to appear on at least two conventional mammograms in standard projection (Sickles, 1994; Maes et al, 1997). Diffuse microcalcifications found in mammograms were eligible if accompanying risk features, such as elongated round or vermiform microcalcifications, were detected. All mammograms were reviewed by the reference radiologist (GW), who was unaware of the results of the histological examination and flow cytometry. Twenty-two healthy women with normal mammographic findings served as controls. The study was approved by the institutional advisory board (ethics committee) of the University of Vienna Medical School and informed consent was obtained from all patients before enrolment in the study.

The preoperative localization was determined with a stereotactically positioned guidewire put in place by the radiologist on the day of surgery. All biopsy specimens were immediately subjected to histological analysis in frozen sections and subsequently embedded in paraffin and stained with haematoxylin and eosin. Histological grading was performed according to Bloom and Richardson (1957). With invasive cancer patients, the axillary lymph nodes (level I and II) were removed. The steroid receptor content was determined immunohistochemically (Stierer et al, 1995).

Before surgery, 10 ml of EDTA-anticoagulated venous blood were drawn to determine the lymphocyte count in peripheral blood (Sysmex NE8000, Toa, Medical Electronics Co. Ltd, Kobe, Japan). Heparinized peripheral blood was used to separate lymphocytes for the investigation by flow cytometry.

**Table 1** Radiological criteria in mammography

| |
|---|
| Star-shaped density |
| Lobulated density |
| Round density |
| Spiculated masses and/or clustered microcalcifications |
| Microcalcifications |
| Number of microcalcifications > 5 $mm^{-2}$ |
| Irregularity in size and/or density |
| Aligned and/or arborescent arrangement |

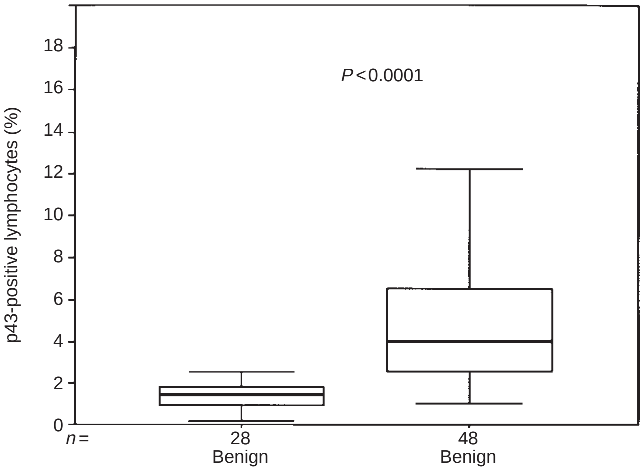

**Figure 1** Box plot depicting the median, interquartile range, as well as the minimum and maximum values of p43 (median) on PBMNC of patients with benign and malignant breast lesions. The median is depicted by the horizontal bars, the interquartile range by the boxes and the minimum and maximum values by the vertical bars. Benign: 1.43% (0.17–3.7), cancer: 3.83% (0.98–19.45), $P$<0.0001, Wilcoxon rank test

**Table 2** Patients' characteristics, prognostic factors and per cent p43-positive lymphocytes

| | *n* | Percentage of p43-positive lymphocytes (median) | Percentage of p43-positive lymphocytes (range) | *P*-value |
|---|---|---|---|---|
| Benign histology | 28 | 1.43 | 0.17–3.7 | |
| Cancer | 48 | 3.83 | 0.98–19.45 | < 0.0001[a] |
| DCIS | 12 | 4.33 | 2.3–19.0 | |
| Invasive | 36 | 3.78 | 0.98–19.45 | |
| Lymph node | | | | |
| Negative | 33 | 3.32 | 0.98–19.45 | |
| Positive | 15 | 5.96 | 1.95–14.03 | 0.042[a] |
| Bloom–Richardson grade | | | | |
| I | 14 | 4.45 | 2.35–14.03 | |
| II | 14 | 3.42 | 0.98–11.98 | |
| III | 20 | 3.31 | 1.19–19.45 | 0.108[a] |
| ER status | | | | |
| Positive | 4 | 3.76 | 0.98–19.02 | |
| Negative | 7 | 6.55 | 3.13–19.45 | 0.068[a] |
| PR status | | | | |
| Positive | 35 | 3.81 | 0.98–14.03 | |
| Negative | 13 | 4.29 | 2.06–19.45 | 0.210[a] |
| Menopausal status | | | | |
| Premenopausal | 12 | 2.85 | 1.0–19.45 | |
| Post-menopausal | 36 | 4.22 | 0.98–19.02 | 0.093[a] |

DCIS = ductal carcinoma in situ; ER = oestrogen receptor; PR = progesterone receptor. [a]Wilcoxon rank test.

© Cancer Research Campaign 1999 

**Table 3** Proportion of patients with p43-positive lymphocytes at different cut-off levels in benign and cancer breast lesions

| | Percentage of p43-positive lymphocytes | | | |
|---|---|---|---|---|
| ***n*** | **0–2** | **2–3** | **3–4** | **>4** |
| Benign (28) | 25 | 3 | 0 | 0 |
| DCIS (12) | 0 | 2 | 3 | 7 |
| Invasive cancer (36) | 4 | 9 | 7 | 16 |

DCIS = ductal carcinoma in situ.

**Table 4** Ratio of total CD4+/CD8+ and p43-positive CD4+/p43-positive CD8+

| Patients' characteristics | Ratio of CD4+/CD8+ median (range) | Ratio of p43+CD4+/p43+CD8+ median (range) | *P*-value |
|---|---|---|---|
| All patients | 2.6 (1.0–9.3) | 1.3 (0.2–18.0) | < 0.001[a] |
| Benign histology | 2.8 (1.0–9.3) | 1.4 (0.2–9.0) | < 0.001[a] |
| Cancer | 2.4 (1.1–7.8) | 1.3 (0.3–18.0) | 0.009[a] |
| DCIS | 2.1 (1.4–4.2) | 2.1 (0.3–18.1) | 0.720[a] |
| Invasive | 2.4 (1.1–7.8) | 1.3 (0.3–18.0) | < 0.001[a] |

DCIS = ductal carcinoma in situ. [a]Wilcoxon rank test.

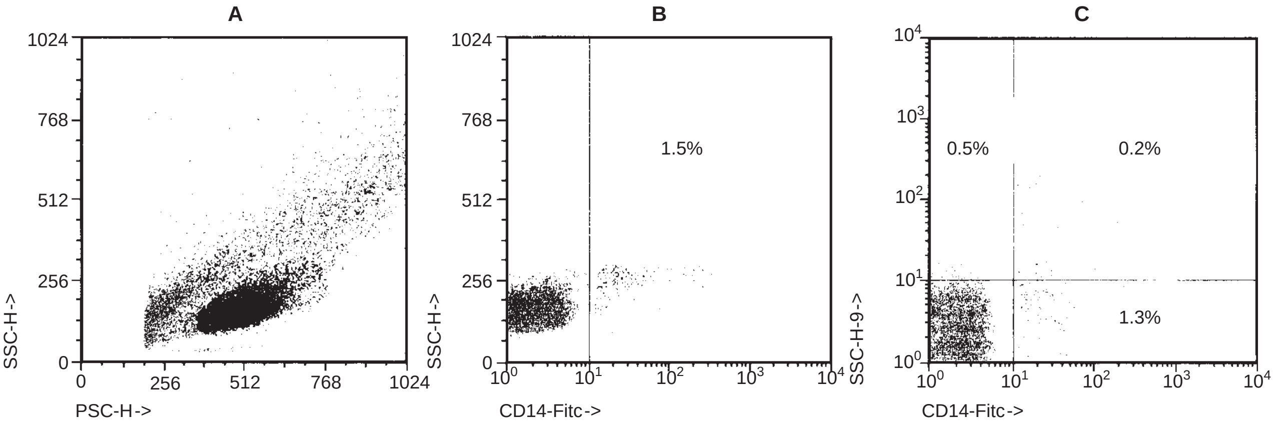

**Figure 2** Forward and side scatter profile of PBMNC from one representative cancer sample. The lymphoid window for analysis was determined with PAINT-A-GATE software (**A**). Three-colour analysis of PBMNC gated for the lymphoid window, CD14 (logarithmic scale) being plotted against side scatter (**B**). Expression of p43 on CD14-positive cells (**C**)

### Flow cytometry

### *Monoclonal antibodies*

The following purified, directly PE and/or PerCP-conjugated monoclonal antibodies (mAbs) and their respective isotype-matched controls were used: LEU-2a (anti-CD8+), LEU-3a (anti-CD4+), LEU-4 (anti-CD3) and LEU-M3 (anti-CD14) (Becton Dickinson, Mountain View, CA, USA). The purified mAb CM-H-9, generously provided by C Moroz, was used for indirect staining with a PE-labelled secondary antibody (DAKO, Santa Barbara, CA, USA).

### Method

Monoclonal and secondary antibodies were titrated for maximum fluorescent intensity. Surface membrane staining of cells, flow cytometry and compensation of the FACSort (Becton Dickinson) were performed as described by other authors (Panzer et al, 1993). Peripheral blood mononuclear cells (PBMNC) were separated by Ficoll-Hypaque density centrifugation. Cells were incubated for 25 min with mAb CM-H-9 on ice, washed once and labelled for another 25 min with the secondary antibody. After a further washing, the cells were incubated for 20 min with anti-CD14-FITC and PerCP anti-CD3, or anti-CD4+, or anti-CD8+. After washing, cells were acquired (30 000 events) using a flow cytometer and analysed with PAINT-A-GATE software (Becton Dickinson). Gating by forward-angle versus right-angle light scatter was performed on the lymphoid window.

### *Statistical methods*

The investigation was performed using the double-blind method, and the histological results were unknown at the time of determination by flow cytometry. Non-parametric tests (Mann–Witney *U*-test, one-way Kruskal–Wallis non-parametric analysis of variance, Wilcoxon matched pairs signed ranks test) were used for univariate comparison of paired parameters. In addition, p43-positive lymphocytes were calculated at different cut-off levels (2%, 3%, and 4% positive cells) to evaluate the sensitivity and specificity of

 © Cancer Research Campaign 1999

this test for prediction of malignancy. The expression of p43 on CD4+ and CD8+ cells was evaluated by determining the ratio between CD4+/CD8+ ratio and p43-positive CD4+/p43-positive CD8+ ratio. A deviation of this ratio from 1 was verified by binomial testing. A significance level of 0.05 was chosen.

### RESULTS

Among the 76 patients with uncertain mammographic findings and non-palpable breast lesions, there were 48 patients with early stage breast cancer, 12 patients with ductal carcinoma in situ (DCIS) and 36 patients with invasive breast cancer. Twenty-eight patients had a benign histology. p43-positive cells in controls were < 2% ($n$ = 22, median 1.3%, range 0.4–1.87). There was a highly significant difference between p43-positive lymphocytes in patients with malignant lesions and those in benign breast tissues ($P$ < 0.0001) (Figure 1). The clinical characteristics and the percentage of lymphocytes expressing p43 in the two groups are shown in Table 2. No difference in prognostic factors, such as lymph node status, grading, menopausal status and steroid receptor status, was observed.

In initial experiments we saw that, among the mononuclear cells, a large population of CD14+ cells (monocyte macrophage population) was stained with the mAb CM-H-9, irrespective of the type of lesion. Therefore, we carefully gated on the lymphoid window, and CD14+ cells were removed from the calculation with the aid of the PAINT-A-GATE software (Figure 2). Subsequently, the expression of p43 on CD3+ cells and on the CD4+ or CD8+ T-cell subpopulation was determined by triple-colour analysis (Figure 3).

Analysis of the sensitivity and specificity at different cut-off levels (2%, 3% and 4% p43-positive cells) revealed a sensitivity of 91.7% and a specificity of 89.3% at 2%, a sensitivity of 68.6% and specificity of 96.4% at 3%, and a sensitivity of 47.9% and specificity of 100% at 4% p43-positive lymphocytes respectively (Table 3).

Of the three patients with a false positive result at a cut-off level of 2%, two had a family history of breast cancer in a first-degree relative. Of the four cancer patients with a p43 result below 2%, two patients had lymph node-positive and grade III breast cancer, respectively, while one patient with 1.96% p43-positive cells had T1a breast cancer.

The ratio among CD4+ and CD8+ cells, p43-positive CD4+ and p43-positive CD8+ is shown in Table 4. There was no significant difference between patients with breast cancer and those with benign lesions. However, the ratio of CD4+/CD8+ was 2.6 (range 1.0–9.3) while the ratio of p43-positive CD4+/p43-positive CD8+ was only 1.3 (range 0.2–18.0) ($P$ < 0.001), thus indicating a significant association between p43 and CD8+ cells. Of interest, there was no significant difference between the ratio of CD4+/CD8+ cells and the ratio of p43-positive CD4+/p43-positive CD8+ cells ($P$ = 0.720) in women with DCIS.

### DISCUSSION

In this study we showed that there was a significantly higher expression of p43 on peripheral lymphocytes in patients with early breast cancer than in women with benign breast lesions. In the peripheral blood of patients with early breast cancer the ratio of CD4+/CD8+ and p43-positive cells shifted in favour of the CD8+ subpopulation.

In an earlier investigation of a large patient group of more than 3000 women, Moroz showed that 80% of stage I and II breast cancer patients had an elevated number of p43-positive lymphocytes as determined by radioimmunoassay (Moroz et al, 1989). These data were confirmed in an unselected group of breast cancer patients by flow cytometry (Rosen et al, 1993). Our aim was to determine whether the expression of p43 might serve as a marker of malignancy in patients with radiologically uncertain findings and non-palpable breast lesions.

At a cut-off level of 2% p43-positive lymphocytes, we achieved a sensitivity of 91.7% and a specificity of 89.3%. False negative p43 values were explained by the presence of highly undifferentiated (grade III) tumours and by lymph node metastases. Likewise, in 122 patients with breast cancer, the expression of p43 was significantly higher in tumour cytosols with grade I or low-grade nuclear pleomorphism than in grade III tumours (Rosen et al, 1992).

We found false positive p43 values in less than 10% of women with benign breast lesions. In a recent publication by Moroz, long-term follow-up of false positive women revealed that elevated p43 was a significant predictor of early breast cancer increasing the normal risk for breast cancer at a factor of about 2.5 (Moroz et al, 1997). It is interesting to note that, in our study, two out of three patients with a false positive p34 result (at a cut-off level of 2%) had a family history of breast cancer in a first-degree relative.

Physiologically, the placental production of p43 may play a role in the prevention or rejection of the embryo by suppression of maternal lymphocytes (Sirota et al, 1989; Maymon and Moroz,

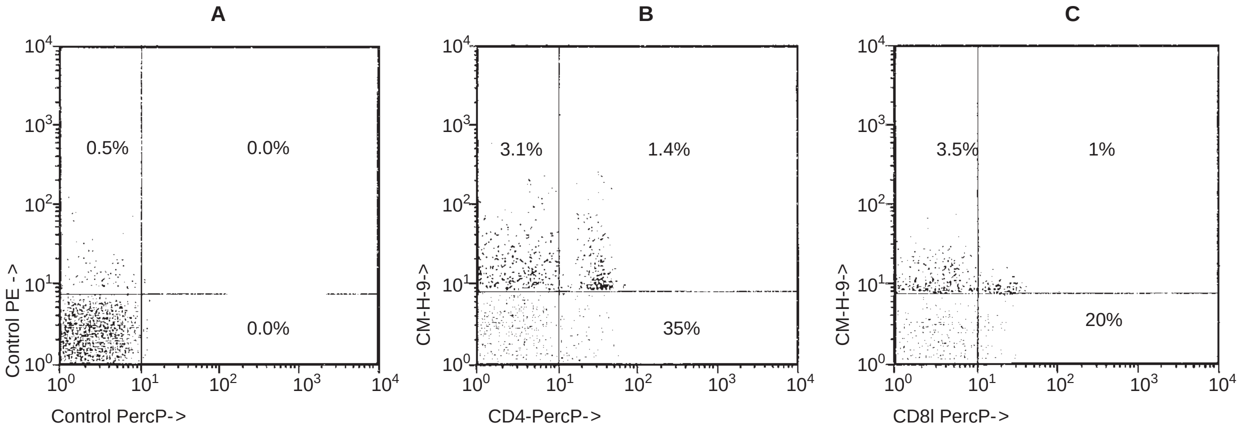

**Figure 3** Three-colour analysis of PBMNC. Experimental design as in Figure 2. Isotype-matched controls for CM-H-9 (p43-PE) and anti-CD4, CD8 PercP (**A**). Expression of p43 on CD4 and CD8 cells (**B**, **C**)

1996). In line with these in vivo observations, it has been demonstrated that p43 has suppressing activation on mitogen-induced lymphocyte proliferation (Fargion et al, 1991). Likewise, increasing amounts of p43 may also be important for T-cell incompetence in patients with HIV infection (Moroz et al, 1989*b*).

The biologic activity of p43 in breast cancer is still not completely understood. It has been shown that this protein is synthesized by placenta, breast cancer tissue and activated T-cells (Garty et al, 1995; Moroz et al, 1997). We can therefore assume that the elevated p43 expression originates from the activated T-cells (reacting against the cancer) and from malignant cells. Consecutively, p43 may provide help to the malignancy, either by induction of autoproliferation or by suppressing anticancer reactive T-cells. The first hypothesis is currently tested by determination of p43 levels after surgical removal of the cancer. We expect a decline of p43 expression after surgical removal.

There is some evidence from our results that, in the CD4+ and CD8+ T-cell subpopulations, CD8+ cells are the major group expressing p43 in breast cancer patients. Their activation to become a cytolytic agent requires help, provided mainly by interleukin 2 (IL-2) from CD4+ cells and autocrine IL-2, and secondary signalling, via CD2 for example (Bach et al, 1989; Meuer et al, 1983). It is interesting to note that the inhibitory effect of p43 on T-cells appears to be mediated by masking CD2 (Maymon and Moroz, 1996; Moroz et al, 1977). The binding of p43 to a particular subset of CD8+ lymphocytes in patients with invasive tumour, but not in those with DCIS, suggests that the affected CD8+ subpopulation plays a specific role in the spread of the tumour. We are focusing now on the characterization of these CD8+ cells to better understand their role for the tumour biology.

In summary, we have shown that a significantly higher proportion of p43-positive peripheral blood lymphocytes can be found in women with early, subclinical breast cancer than in women with benign breast lesions. The determination of p43 in peripheral blood could serve as an additional tool in the diagnosis of women who present with controversial mammographic findings. It could also reduce the cost of intensive, and often uncomfortable, further investigation.

### ACKNOWLEDGEMENTS

We are grateful to Chaya Moroz from the Department of Molecular Immunology, Rabin Medical Centre, Beilinson Campus, Petah Tikva (Israel) for providing the monoclonal antibody CM-H-9 and for her invaluable discussion of our manuscript. This study was supported by grant 6263 from the funds for scientific research by the Österreichische Nationalbank.

### REFERENCES

Arnesson L, Vitak B, Manson J, Fagerberg G and Smeds S (1995) Diagnostic outcome of repeated mammography screening. *World J Surg* **19**: 372–377

Bach F, Geller R, Nelson P, Panzer S, Gromo G, Benfield M, Inverardi L, Podack E, Witson J and Houchins J (1989) A ‘minimal signal-stepwise activation’ analysis of functional maturation of T lymphocytes. *Immunol Rev* **111**: 35–57

Bilik R, Mor C, Hazaz B and Moroz C (1989) Characterization of T-lymphocyte subpopulations infiltrating primary breast cancer. *Cancer Immunol Immunother* **28**: 143–147

Black W, Nease R and Tosteson A (1995) Perceptions of breast cancer risk and screening effectiveness in women younger than 50 years of age. *J Natl Cancer Inst* **87**: 720–731

Bloom H and Richardson W (1957) Histological grading and prognosis in breast cancer. *Br J Cancer* **11**: 259–364

Ciatto S, Rosselli-Del T and Zappa M (1995) The detectability of breast cancer by screening mammography. *Br J Cancer* **71**: 337–339

Fargion S, Fracanzani A, Bando B, Arosio P, Levi S and Fiorelli G (1991) Specific binding sites of H-ferritin on human lymphocytes modulation during cellular proliferation and potential implication in cell growth control. *Blood* **78**: 1056–1061

Flanagan F, Dehdashti F and Siegel B (1998) PET in breast cancer. *Semin Nucl Med* **28**: 290–302

Friedrich M (1998) MRI of the breast: state of the art. *Eur Radiol* **8**: 707–725

Garty B, Kaminsky E and Moroz C (1995) The immunosuppressive human placental ferritin subunit p43 is produced by activated CD4+ lymphocytes. *Clin Diagn Lab Immunol* **2**: 225–226

Harris R and Leininger L (1995) Clinical strategies for breast cancer screening: weighing and using the evidence. *Ann Intern Med* **122**: 539–547

Hiatt R and Pasick R (1996) Unsolved problems in early breast cancer detection: focus on the underserved. *Breast Cancer Res Treat* **40**: 37–51

Leitch A (1995) Controversies in breast cancer screening. *Cancer* **76**: 2064–2069

Lidbrink E, Elfving J, Frisell J and Jonsson E (1996) Neglected aspects of false positive findings of mammography in breast cancer screening: analysis of false positive cases from the Stockholm trial. *Br Med J* **312**: 273–276

Maes R, Dronkers D, Hendriks J, Thijssen M and Nab H (1997) Do non-specific minimal signs in a biennial mammographic breast cancer screening programme need further diagnostic assessment? *Br J Radiol* **70**: 34–38

Maymon R and Moroz C (1996) Placental isoferritin: a new biomarker from conception to delivery. *Br J Obstet Gynaecol* **103**: 301–305

Meuer S, Cooper D, Hodgdon J, Hussey R, Fitzgerald K, Schlossman S and Reinherz E (1983) Identification of the receptor for antigen and major histocompatibility complex on human inducer T lymphocytes. *Science* **222**: 1239–1242

Moroz C, Giler S, Kupfer B and Urca I (1977) Lymphocytes bearing surface ferritin in patients with Hodgkin’s disease and breast cancer. *N Engl J Med* **298**: 1175–1176

Moroz C, Kupfer B, Twig S and Parhami S (1985) Preperation and characterization of monoclonal antibodies specific to placenta ferritin. *Clin Chim Acta* **148**: 111–118

Moroz C, Kahn M, Ron E, Luria H and Chaimoff C (1989*a*) The use of oncofetal ferritin-bearing lymphocytes as a marker for the screening, diagnosis, and follow-up of patients with early breast malignancy screening of 3400 women. *Cancer* **64**: 691–697

Moroz C, Misrock S and Siegal F (1989*b*) Isoferritins in HIV infection: relation to clinical stage and the pathogenesis of AIDS. *AIDS* **3**: 11–16

Moroz C, Chetrit A, Kahn M and Modan B (1997) FBL blood test as a predictive marker of breast cancer in high risk women. *Med Oncol* **14**: 39–42

Panzer S, Madden M and Matsushi M (1993) Interaction of IL-1, IL-6 and tumour necrosis factor-alpha (TNF-$\alpha$) in human T cells activated by murine antigen. *Clin Exp Immunol* **93**: 471–478

Reinerova M, Rosen H, Veselovska Z, Stierer M and Svec J (1993) Expression of p43 associated placental isoferritin (PLF) correlates inversely with cell growth in breast cancer cell lines MCF-7 and T47-D. *Neoplasma* **40**: 147–151

Rosen H, Moroz C, Reiner A, Reinerova M, Stierer M, Svec J, Schemper M and Jakesz R (1992) Placental isoferritin associated p43 antigen correlates with features of high differentiation in breast cancer. *Breast Cancer Res Treat* **24**: 17–26

Rosen H, Stierer M, Gottlicher J, Wolf H, Spoula H and Eibl M (1993) Determination of placental ferritin-positive peripheral lymphocytes in early stages of breast cancer. *Am J Surg* **165**: 213–217

Rosen H, Ausch C, Reiner G, Reinerova M, Svec J, Tuchler H, Schiessel R and Moroz C (1996) Immunosuppression by breast cancer associated p43 – effect of immunomodulators. *Breast Cancer Res Treat* **41**: 171–176

Sedlak J, Reinerova M, Hunakova L, Ausch C, Chorvath B, Rosen H and Moroz C (1995) Alterations of cell surface antigens induced by placental isoform of ferritin in human carcinoma cell lines. *Cancer Lett* **94**: 101–106

Sickles E (1994) Non-palpable, circumscribed, non-calcified solid breast masses: likelihood of malignancy based on lesion size and age of patient. *Radiology* **192**: 439–443

Sirota L, Kupfer B and Moroz C (1989) Placental isoferritin as a physiological downregulator of cellular immunoreactivity during pregnancy. *Clin Exp Immunol* **77**: 257–262

Stierer M, Rosen H, Weber R, Hanak H, Auerbach L, Spona J and Tuchler H (1995) A prospective analysis of immunohistochemically determined hormone receptors and nuclear features as predictor of early recurrence in primary breast cancer. *Breast Cancer Res Treat* **36**: 11–21

Tabar L, Fagerberg G, Chen H, Duffy S, Smart C, Gad A and Smith R (1995) Efficacy of breast cancer screening by age. New results from the Swedish Two-County Trial. *Cancer* **75**: 2507–2517

© Cancer Research Campaign 1999
